# Transcriptome analysis reveals a long non-coding RNA signature to improve biochemical recurrence prediction in prostate cancer

**DOI:** 10.18632/oncotarget.25048

**Published:** 2018-05-18

**Authors:** Jinyuan Xu, Yujia Lan, Fulong Yu, Shiwei Zhu, Jianrong Ran, Jiali Zhu, Hongyi Zhang, Lili Li, Shujun Cheng, Yun Xiao, Xia Li

**Affiliations:** ^1^ College of Bioinformatics Science and Technology, Harbin Medical University, Harbin, Heilongjiang 150086, China; ^2^ State Key Laboratory of Molecular Oncology, Department of Etiology and Carcinogenesis, Cancer Institute and Hospital, Peking Union Medical College and Chinese Academy of Medical Sciences, Beijing 100021, China

**Keywords:** lncRNA signature, prostate cancer, transcriptome, prognosis, BCR-free survival

## Abstract

Despite highly successful treatments for localized prostate cancer (PCa), prognostic biomarkers are needed to improve patient management and prognosis. Accumulating evidence suggests that long noncoding RNAs (lncRNAs) are key regulators with biological and clinical significance. By transcriptome analysis, we identified a set of consistently dysregulated lncRNAs in PCa across different datasets and revealed an eight-lncRNA signature that significantly associated with the biochemical recurrence (BCR)-free survival. Based on the signature, patients could be classified into high- and low-risk groups with significantly different survival (HR = 2.19; 95% CI = 1.67–2.88; *P* < 0.0001). Validations in the validation cohorts and another independent cohort confirmed its prognostic value for recurrence prediction. Multivariable analysis showed that the signature was independent of common clinicopathological features and stratified analysis further revealed its role in elevating risk stratification of current prognostic models. Additionally, the eight-lncRNA signature was able to improve on the CAPRA-S score for the prediction of BCR as well as to reflect the metastatic potential of PCa. Functional characterization suggested that these lncRNAs which showed PCa-specific expression patterns may involve in critical processes in tumorigenesis. Overall, our results demonstrated potential application of lncRNAs as novel independent biomarkers. The eight-lncRNA signature may have clinical potential for facilitating further stratification of more aggressive patients who would benefit from adjuvant therapy.

## INTRODUCTION

Prostate cancer (PCa) remains the most common cancer and a leading cause of cancer-related death in men, with the most new patients diagnosed with the disease last year [1]. Despite the majority of prostate cancer patients are diagnosed at a potentially curable stage and treated with radical prostatectomy or other first-line treatments, a subset of patients will experience a recurrence, typically detected by a rise in serum prostate-specific antigen (PSA) levels [2, 3]. Biochemical recurrence (BCR) is a well-established predictor of clinical recurrence and metastasis of PCa, which is widely used as an early end point to define the treatment success. The ability to predict the risk of BCR soon after surgery could allow for initiation of secondary therapy as necessary to improve long term treatment outcomes [4, 5]. However, current clinicopathological models, which incorporate parameters such as PSA concentration, Gleason score and positive lymph nodes, are insufficient to accurately define BCR across all treatment modalities [6, 7]. Therefore, the prediction of the likelihood of BCR is critical for surveillance strategy of PCa patients and the molecular underpinnings of aggressive and indolent cancers may be essential to improve patient management and prognosis [8].

Recent studies demonstrated the utility of gene expression-based signatures in the prognosis [9, 10]. Currently, the roles of the long non-coding RNAs (lncRNAs) in human cancers have received considerable attention [11, 12]. Accumulating evidences suggest that lncRNAs are frequently aberrantly expressed in cancers and typically exhibit tissue-specific expression patterns, which may be essential players in tumorigenesis [13–15]. Moreover, lncRNA expression may confer clinical information about disease outcomes and have utility as biomarkers in diagnosis and prognostication [16, 17]. In PCa, the lack of appropriate sensitive and specific biomarkers makes lncRNAs promising novel biomarkers as well as therapeutic targets for the disease [18–20]. Thus, exploring a lncRNA signature for diagnosis and risk stratification would be valuable to improve management of PCa patients.

Here, by using large independent patient cohorts, we identified an eight-lncRNA signature with the ability to predict the BCR of patients with PCa and demonstrated that the lncRNA signature could be used as an independent prognostic factor and improve risk stratification of current prognostic models.

## RESULTS

### Transcriptome analysis reveals consistently dysregulated lncRNAs in three PCa cohorts

Patients with pathologically confirmed PCa and corresponding clinical data from the Cancer Genome Atlas (TCGA) were included in our study. After removal of patients without recurrence status a total of 307 patients were recruited and randomly assigned into training (184 patients) and validation (123 patients) sets. Based on the transcriptome profiles of 50 paired PCa and matched adjacent normal tissues from the training set, we identified 2,906 lncRNAs that were differentially expressed (FDR < 0.05 and fold change > 1.2). To further extract a core lncRNA gene set related to clinical outcomes in patients with PCa, we performed an integrative transcriptome analysis using another two cohorts: (1) transcriptome sequencing of 14 primary tumors and adjacent normal pairs from Ren *et al.* [21] and (2) exon arrays of 47 primary and 48 normal tissues from Brase *et al.* [22]. We identified 1522 and 2410 differentially expressed genes between tumors and control for these two cohorts, respectively (FDR < 0.05 and fold change > 1.2, see Materials and methods). Across all three cohorts, 142 lncRNAs were found to be consistently altered, which were used in the following analysis (Supplementary Figure 1 and Supplementary Table 1).

### Identification of an eight-lncRNA signature associated with BCR-free survival

By subjecting the expression levels of the consistently altered lncRNAs of the training cohort to univariate Cox regression analysis, we identified eight lncRNAs that were significantly related to BCR-free survival (Table 1, *P* < 0.05). Among the eight lncRNAs, the higher expression of one lncRNA (AC005632.2) was strongly associated with longer BCR-free survival, whereas for the others (PCAT7, SLC12A9-AS1, RGMB-AS1, PCAT1, AP002992.1, AC025265.1, LINC00593), the higher expression of them were associated with shorter BCR-free survival. Based on the risk score formula (see Methods), the eight-lncRNA signature risk score for each patient in the training cohort was computed. Patients were divided into a high-risk group (*n* = 92) and a low-risk group (*n* = 92) using the median of risk scores as the cutoff point.

**Table 1:** LncRNAs significantly associated with the BCR-free survival in the training cohort (*n* = 184)

Gene symbol	Chromosomal position	*P* value	Hazard ratio (95% CI)	Coefficient
PCAT7	chr9:94555069–94568127	0.009	1.58 (1.12–2.22)	0.45666
SLC12A9-AS1	chr7:100837314–100852616	0.025	1.30 (1.03–1.63)	0.26201
RGMB-AS1	chr5:98769618–98773469	0.041	1.10 (1.004–1.22)	0.09976
PCAT1	chr8:126847055–127021014	0.006	1.17 (1.05–1.32)	0.16082
AP002992.1	chr11:68122053–68130518	0.0003	3.39 (1.74–6.58)	1.22014
AC025265.1	chr12:103746315–103768858	0.016	1.58 (1.09–2.30)	0.45910
LINC00593	chr15:69835234–69843120	0.028	5.10 (1.19–21.84)	1.62870
AC005632.2	chr16:21626742–21627569	0.025	0.003 (0.000019–0.49)	–5.80340

*P*-values are calculated from the univariable Cox regression analysis.

### Prognostic value of the eight-lncRNA signature in the training, validation and combined sets

**Figure 1:**
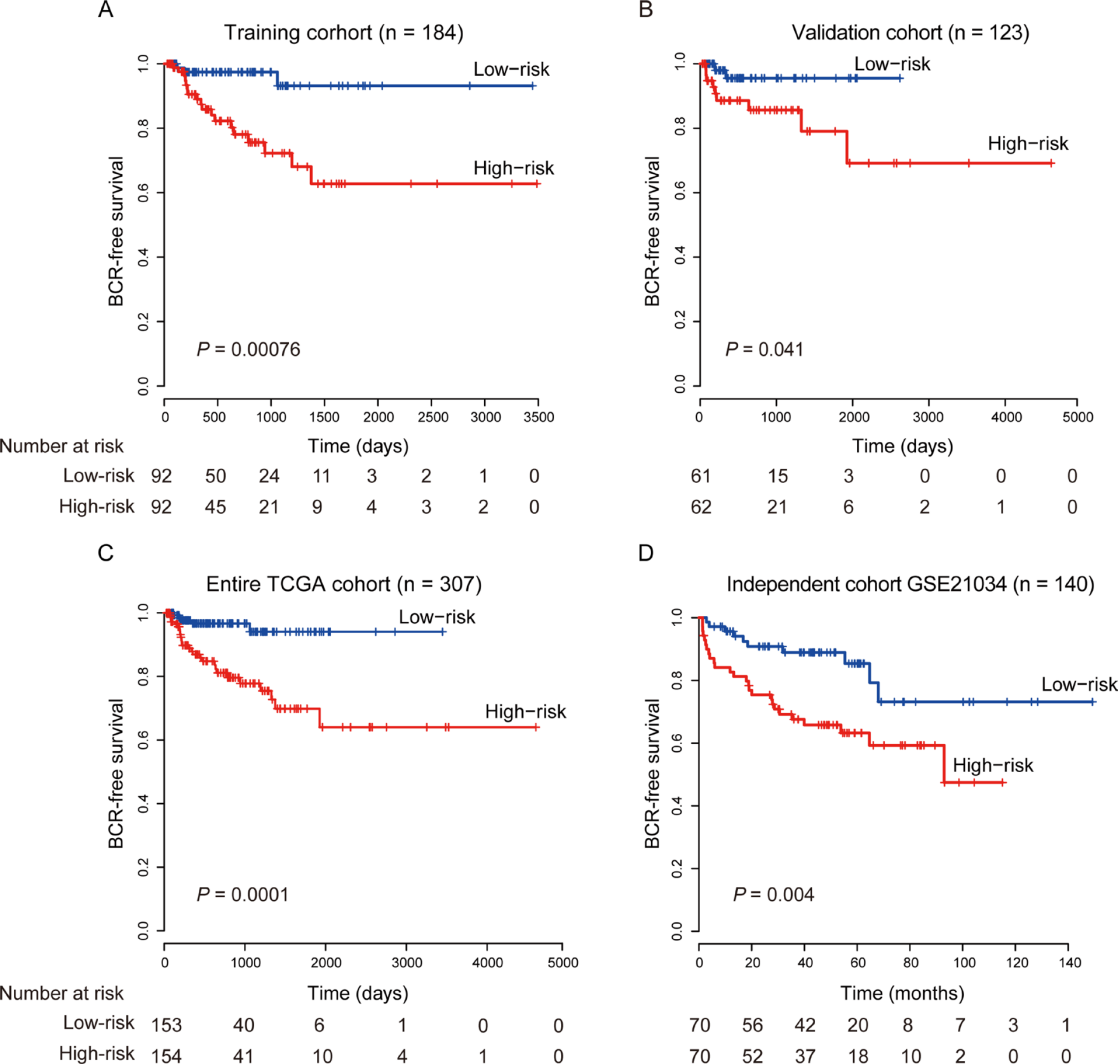
Kaplan–Meier estimates of the BCR-free survival of PCa patients using the eight-lncRNA signature (**A**) Training cohort (*n* = 184). (**B**) Validation cohort (*n* = 123). (**C**) Entire TCGA cohort (*n* = 307). (**D**) Independent cohort GSE21034 (*n* = 140). The differences between the two curves were determined by the two-side log-rank test.

**Figure 2:**
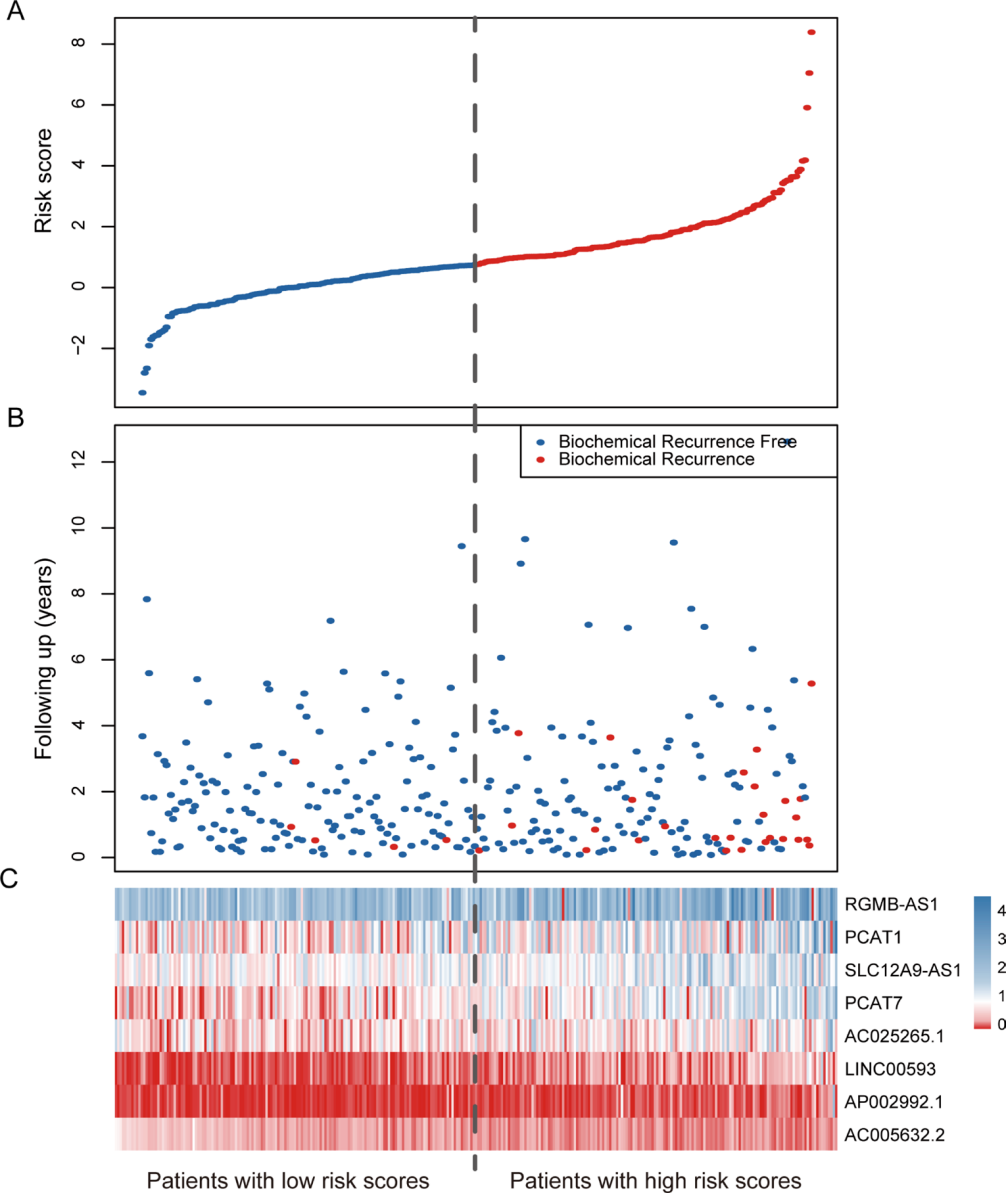
LncRNA risk score analysis of the entire TCGA cohort (**A**) The distribution of the eight-lncRNA risk score. (**B**) Patients’ BCR status and time. (**C**) Heatmap of the eight lncRNA expression profiles. The dotted line represents the median lncRNA risk score cutoff dividing patient into low-risk and high-risk groups.

To evaluate the prognostic effect of the eight-lncRNA signature on BCR-free survival, we first used Kaplan-Meier analysis to examine the differences in BCR-free survival of the PCa patients in the high-risk and low-risk groups. In the training cohort, patients in the high-risk group had shorter BCR-free survival time than patients in the low-risk group (Figure 1A, log-rank test, *P* = 0.0007). By using the same risk formula, patients in the validation cohort (*n* = 123) and the entire TCGA PCa cohort (*n* = 307) were classified into high-risk or low-risk group and similar results were also found (validation cohort: Figure 1B, log-rank test, *P* = 0.041; entire TCGA PCa cohort: Figure 1C, log-rank test, *P* = 0.0001). In consistence with the findings described above, univariate Cox regression analysis also found that patients with a high eight-lncRNA risk score had significantly shorter BCR-free survival time than patients with a low score in all three cohorts (training cohort: HR = 2.19, 95% CI = 1.67–2.88, *P* < 0.0001; validation cohort: HR = 1.37, 95% CI = 1.09–1.71, *P* = 0.006; entire TCGA PCa cohort: HR = 1.51, 95% CI =1.32–1.72, *P* < 0.0001) (Table 2). By investigating the distribution of the eight-lncRNA risk score, patients’ survival status and the lncRNA expression, we showed that PCa patients with high prognostic scores tended to have BCR and express high levels of risky lncRNAs (PCAT7, SLC12A9-AS1, RGMB-AS1, PCAT1, AP002992.1, AC025265.1, LINC00593), whereas those with low scores tended to express the protective lncRNA (AC005632.2) (Figure 2).

**Table 2:** Univariate and multivariate Cox regression analysis in the training, validation and entire cohorts

Predictors	Univariate analysis		Multivariate analysis	
	HR (95% CI)	*p* value	HR (95% CI)	*p* value
Training cohort (*N* = 184)				
Risk score	2.19 (1.67–2.88)	<0.0001	2.19 (1.49–3.22)	<0.0001
Age, yr	1.03 (0.96–1.10)	0.48	1.08 (0.96–1.21)	0.21
Gleason score		0.02		0.06
≤7	1.00 (Ref.)		1.00 (Ref.)	
≥8	2.82 (1.19–6.72)		3.298 (0.94–11.596)	
T stage		0.01		0.75
T2a-c	1.00 (Ref.)		1.00 (Ref.)	
T3a-c + T4	6.59 (1.53–28.36)		1.33 (0.23–7.78)	
N stage		0.03		0.57
N0	1.00 (Ref.)		1.00 (Ref.)	
N1	3.00 (1.14–7.92)		1.42 (0.43–4.70)	
Lymph Node(s) Examined Number	1.05 (1.02–1.09)	0.003	1.04 (0.997–1.09)	0.068
Positive lymph node		0.01		NA
0	1.00 (Ref.)		1.00 (Ref.)	
>0	3.60 (1.30–9.95)		NA	
Surgical margin status		0.67		0.588
Negative	1.00 (Ref.)		1.00 (Ref.)	
Positive	1.23 (0.48–3.17)		1.44 (0.386–5.366)	
AKT1	0.65 (0.195–2.14)	0.47	2.19 (0.03–3.24)	0.33
BCL2	0.857 (0.55–1.34)	0.499	2.04 (1.01–4.098)	0.046
CCL4	0.797 (0.577–1.099)	0.166	0.84 (0.498–1.42)	0.52
KLK2	1.65 (0.785–3.47)	0.186	2.66 (0.82–8.69)	0.105
Validation cohort (*N* = 123)				
Risk score	1.37 (1.09–1.71)	0.006	1.67 (1.06–2.63)	0.027
Age, yr	1.01 (0.93–1.10)	0.75	1.03 (0.93–1.14)	0.61
Gleason score		0.004		0.035
≤7	1.00 (Ref.)		1.00 (Ref.)	
≥8	9.25 (2.00–42.87)		7.076 (1.14–43.78)	
T stage		0.09		0.67
T2a-c	1.00 (Ref.)		1.00 (Ref.)	
T3a-c + T4	3.79 (0.81–17.78)		1.58 (0.19–13.34)	
N stage		0.04		0.03
N0	1.00 (Ref.)		1.00 (Ref.)	
N1	3.65 (1.06–12.57)		6.91 (1.15–41.42)	
Lymph Node(s) Examined Number	1.02 (0.97–1.06)	0.37	0.987 (0.92–1.06)	0.71
Positive lymph node		0.047		NA
0	1.00 (Ref.)		1.00 (Ref.)	
>0	3.48 (1.01–11.98)		NA	
Surgical margin status		0.64		0.03
Negative	1.00 (Ref.)		1.00 (Ref.)	
Positive	0.72 (0.19–2.75)		0.096 (0.012–0.77)	
AKT1	0.84 (0.11–6.39)	0.865	0.65 (0.051–8.29)	0.74
BCL2	0.76 (0.41–1.41)	0.38	0.61 (0.24–1.56)	0.30
CCL4	0.82 (0.50–1.35)	0.44	0.94 (0.47–1.91)	0.87
KLK2	0.65 (0.28–1.45)	0.29	0.21 (0.055–0.83)	0.026
Entire cohort (*N* = 307)				
Risk score	1.51 (1.32–1.72)	<0.0001	1.61 (1.24–2.07)	0.0003
Age, yr	1.02 (0.97–1.08)	0.48	1.04 (0.98–1.11)	0.17
Gleason score		0.0002		0.07
≤7	1.00 (Ref.)		1.00 (Ref.)	
≥8	4.10 (1.97–8.50)		2.25 (0.93–5.47)	
T stage		0.002		0.28
T2a-c	1.00 (Ref.)		1.00 (Ref.)	
T3a-c + T4	5.27 (1.84–15.08)		1.97 (0.58–6.77)	
N stage		0.003		0.03
N0	1.00 (Ref.)		1.00 (Ref.)	
N1	3.18 (1.49–6.81)		2.78 (1.10–7.07)	
Lymph Node(s) Examined Number	1.03 (1.01–1.06)	0.008	0.994 (0.96–1.03)	0.75
Positive lymph node		0.002		NA
0	1.00 (Ref.)		1.00 (Ref.)	
>0	3.50 (1.60–7.65)		NA	
Surgical margin status		0.90		0.03
Negative	1.00 (Ref.)		1.00 (Ref.)	
Positive	0.95 (0.44–2.06)		0.35 (0.13–0.92)	
AKT1	0.69 (0.24–1.9995)	0.50	0.899 (0.24–3.30)	0.87
BCL2	0.82 (0.57–1.186)	0.297	1.03 (0.64–1.68)	0.89
CCL4	0.78 (0.597–1.03)	0.082	0.90 (0.63–1.30)	0.59
KLK2	1.09 (0.66–1.81)	0.73	1.04 (0.59–1.86)	0.88

HR, hazard ratio; CI, confidence interval.

### Further validation of the eight-lncRNA signature in another independent PCa cohort

We further validated the prognostic power of the eight-lncRNA signature in another independent PCa cohort from GEO accession GSE21034 (*n* = 140) [23]. According to our risk formula, the patients were classified as high-risk (*n* = 70) or low-risk (*n* = 70) groups. Consistent with the findings in the TCGA cohorts, patients in the high-risk group suffered significantly poor BCR-free survival than those in the low-risk group (Figure 1D, log-rank test, *P* = 0.004). The results in the univariate Cox regression model also showed that the eight-lncRNA signature was significantly associated with BCR-free survival in this independent cohort (Table 2, HR = 1.16, 95% CI = 1.07–1.27, *P* = 0.0005).

### Prognostic value of the eight-lncRNA signature is independent of clinical and pathological factors

To investigate whether the eight-lncRNA signature is an independent predictor of prognosis, additional clinicopathological factors in the TCGA cohort, such as age, Gleason score, T stage, positive lymph node (LN) and four known biomarkers [24–27], were analyzed in a multivariate Cox regression model. The results showed that the eight-lncRNA signature remained to be significantly associated with BCR-free survival in both training and validation cohort, while other factors in the model were not (Table 2, HR = 2.19, 95% CI = 1.49–3.22, *P* < 0.0001 and HR = 1.67, 95% CI = 1.06–2.63, *P* = 0.027, respectively). And the results from the entire cohort showed that the eight-lncRNA signature (HR = 1.61, 95% CI = 1.24–2.07, *P* = 0.0003), as well as N stage (HR = 2.78, 95% CI = 1.10–7.07, *P* = 0.03) and Surgical margin status (HR = 0.35, 95% CI = 0.13–0.92, *P* = 0.03) were independent prognostic factors (Table 2). In the multivariate Cox regression model on the independent cohort, the eight-lncRNA signature, age, T stage, positive LN and CAPRA score, etc., were defined as covariates. We also found that the prognostic power of the eight-lncRNA signature was indeed independent of these clinical features (Table 3, HR =1.20, 95% CI = 1.08–1.33, *P* = 0.00095).

**Table 3:** Univariate and multivariate Cox regression analyses in GSE21034 cohort

Predictors	Univariate analysis		Multivariate analysis	
	HR (95% CI)	*P* value	HR (95% CI)	*P* value
Risk score	1.16 (1.07–1.27)	0.0005	1.20 (1.08–1.33)	0.00095
Age, yr	1.02 (0.97–1.07)	0.43	0.92 (0.86–0.99)	0.026
T stage				
T1a-c	1.00 (Ref.)		1.00 (Ref.)	
T2a-c	0.75 (0.36–1.59)	0.46	0.30 (0.12–0.74)	0.0095
T3a-c	5.05 (1.86–13.73)	0.015	1.96 (0.49–7.80)	0.34
Pathologic tumor stage		<0.0001		0.38
T2a-c	1.00 (Ref.)		1.00 (Ref.)	
T3a-c + T4	5.23 (2.57–10.68)		1.61 (0.55–4.68)	
Lymph nodes examined	0.995 (0.94–1.05)	0.87	0.98 (0.93–1.03)	0.40
Positive lymph nodes		<0.0001		<0.0001
0	1.00 (Ref.)		1.00 (Ref.)	
>0	9.21 (4.44–19.08)		9.40 (3.13–28.19)	
CAPRA-S				
low	1.00 (Ref.)		1.00 (Ref.)	
Intermediate	5.56 (1.84–16.79)	0.002	5.96 (1.71–20.68)	0.005
High	13.03 (4.27–39.75)	<0.0001	7.06 (1.69–29.47)	0.007

### Comparison of the eight-lncRNA signature with Gleason score and positive lymph node

**Figure 3:**
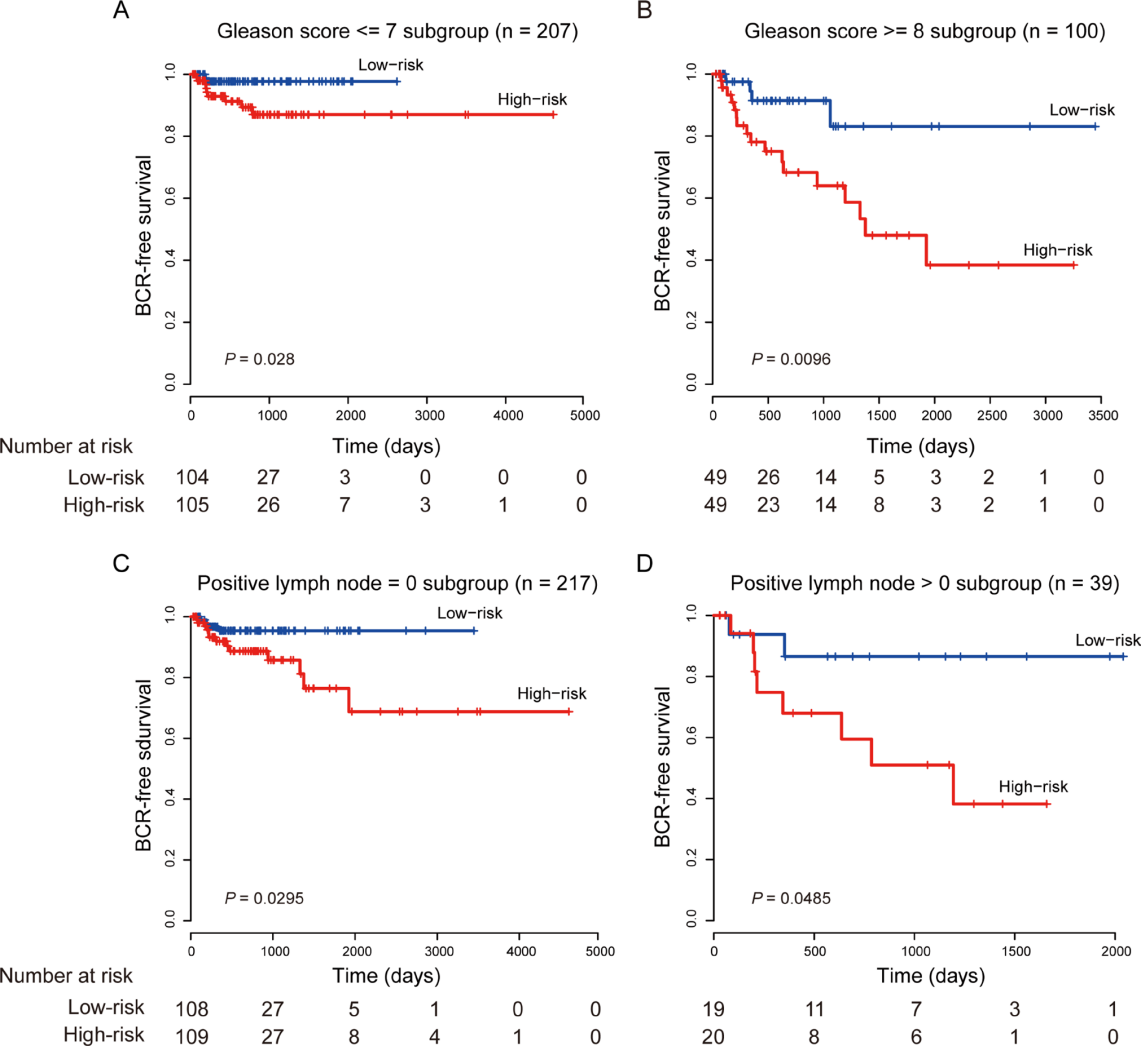
Kaplan–Meier estimates of the BCR-free survival of the entire TCGA cohort stratified by Gleason score and positive lymph node (**A**) Patients with Gleason score ≤ 7. (**B**) Patients with Gleason score ≥ 8. (**C**) Patients with positive LNs = 0. (**D**) Patients with positive LNs > 0.

The Gleason score is a powerful predictor to help evaluate the prognosis of patients with prostate cancer. Patients with a higher Gleason score tend to have more aggressive prostate cancers and have a worse prognosis [28]. Although it is important, Gleason score alone is not sufficiently accurate to predict pathologic stage. So, patients with low-grade (Gleason score 2–7) tumors are still at risk of recurrence, and not all patients with high-grade (Gleason score ≥ 8) prostate cancer will progress to invasive carcinoma [29, 30]. In multivariate survival analysis, we found that the eight-lncRNA signature retained significant prognostic value in all tested cohorts independent of Gleason score. To test weather our lncRNA signature could predict prognosis of patients within the same Gleason grade, a stratified analysis was performed in low- and high-grade Gleason score patients. The stratification analysis showed that the signature could further classify patients into different prognoses. For patients with low-grade (Gleason score 2–7, *n* = 207) tumors, the signature subdivided them into those likely to have longer versus shorter BCR-free survival times (log-rank test, *P* = 0.028, Figure 3A). Similarly, among patients with high-grade (Gleason score ≥ 8, *n* = 100), the signature could also subdivide them into two groups with significantly disparate survival (log-rank test, *P* < 0.01, Figure 3B).

Several studies have shown that patients with lymph node positive prostate cancer indicate an aggressive disease progression and have long BCR-free survival [31–33]. In our study, we also found that our eight-lncRNA signature was independent of positive LN and could effectively stratify patients with different prognoses in conjunction with number of nodes involved (Figure 3C and 3D).

**Figure 4:**
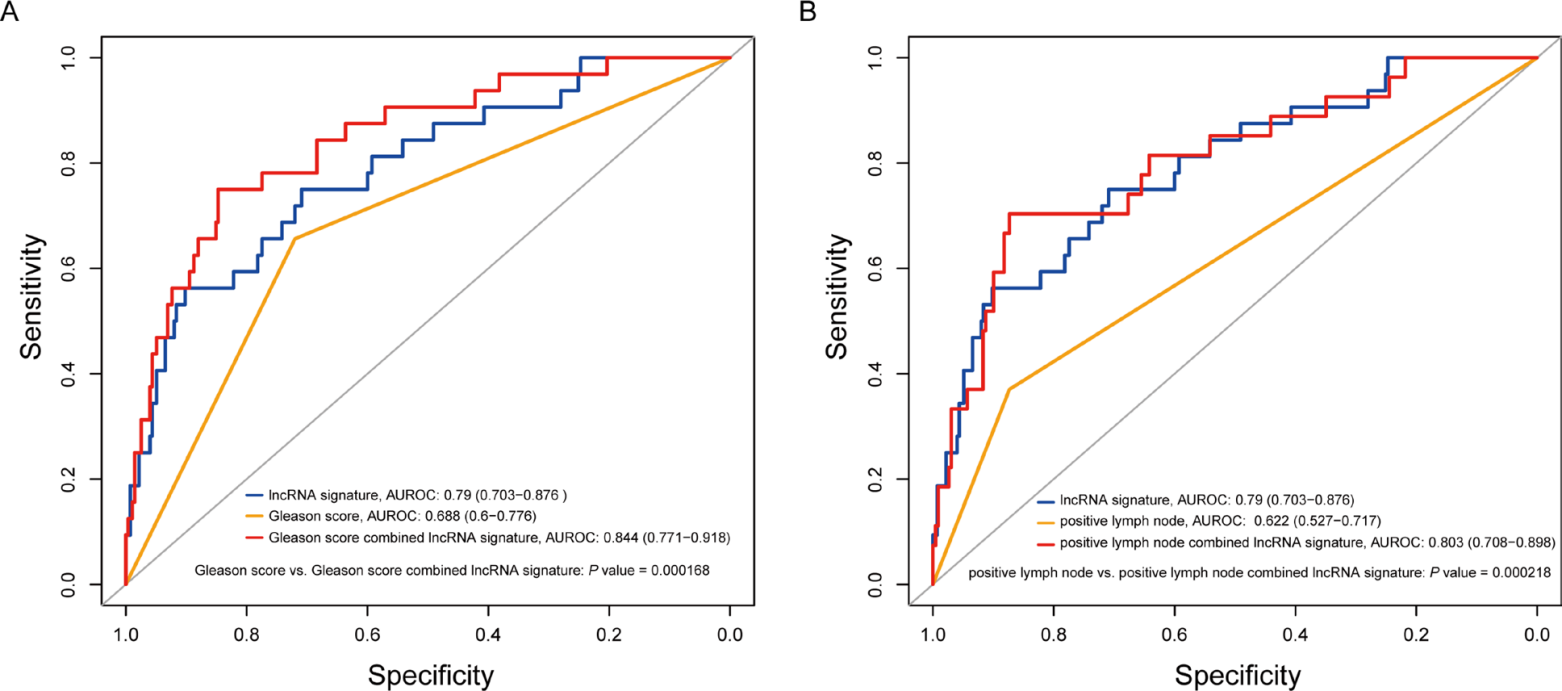
Comparison of sensitivity and specificity for survival prediction by the eight-lncRNA signature, Gleason score and positive lymph node (**A**) ROC curves of the eight-lncRNA signature, Gleason score and the combination of the two factors. (**B**) ROC curves of the eight-lncRNA signature, positive LN and their combination. *P* values showed the AUC of Gleason score versus the AUC of the combination of signature and Gleason score and the AUC of positive LN versus the AUC of the combination of signature and positive LN.

The eight-lncRNA signature displayed superior prognostic accuracy when tested against Gleason score and positive LN. By ROC analysis, the eight-lncRNA signature had a better prognostic ability than both Gleason score and positive LN. And the combinations of the eight-lncRNA signature with Gleason score or positively LN can significantly elevate the prognostic ability of Gleason score or positive LN alone (the area under the curve (AUC), 0.84 vs. 0.69, *P* = 0.00017 and 0.80 vs. 0.62, *P* = 0.00022, respectively) (Figure 4).

### The lncRNA signature can significantly improve the predictive value of the CAPRA-S score and is associated with metastatic progression

**Figure 5:**
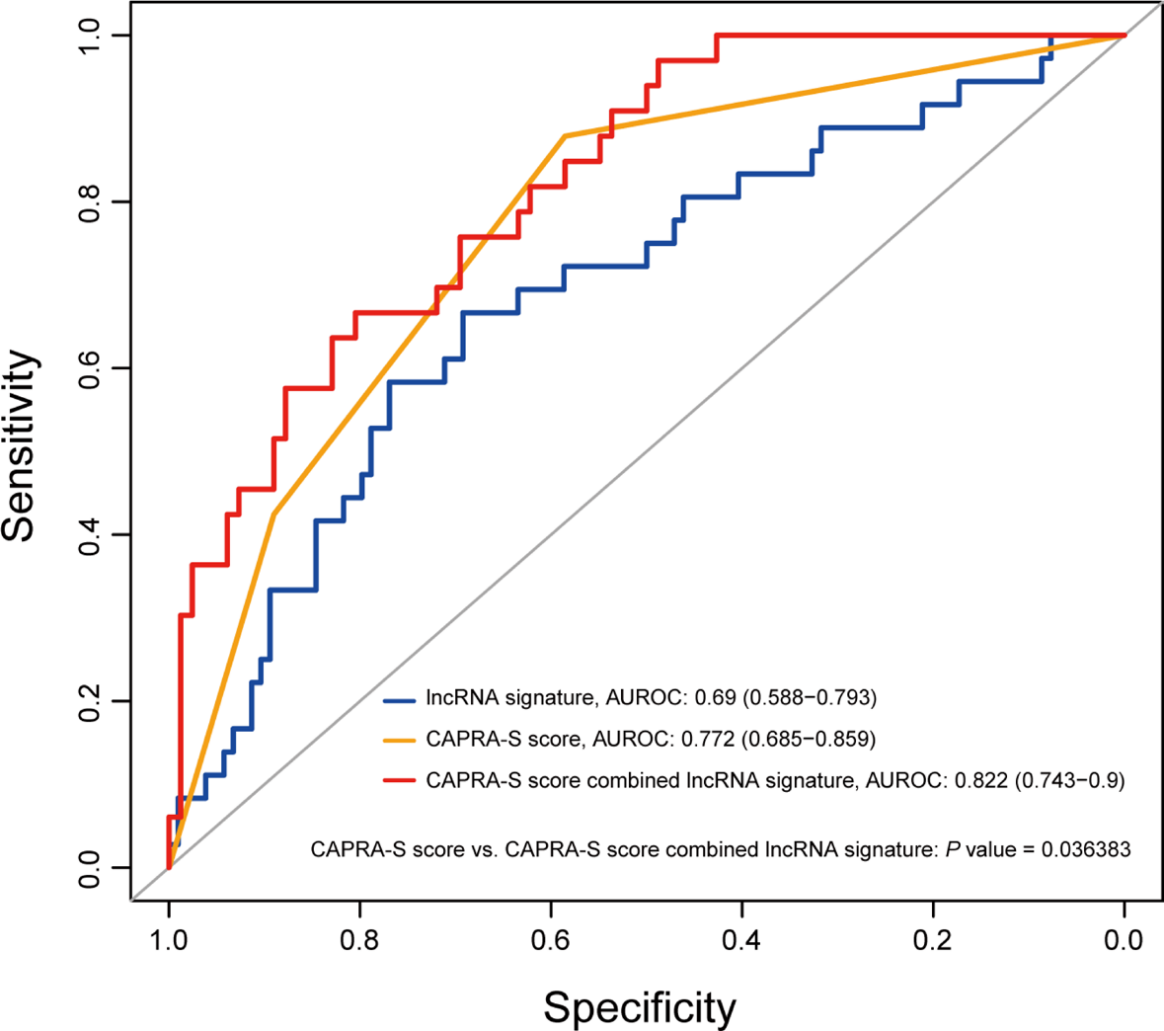
The eight-lncRNA signature significantly improved the prediction value of the CAPRA-S score The prediction capability of the eight-lncRNA risk score, CAPRA-S score and combination of the two factors is evaluated by the ROC curve in GSE21034 cohort. *P* value showed the AUC of CAPRA-S score versus the AUC of the combination of signature and CAPRA-S score.

The cancer of the prostate risk assessment post-surgical (CAPRA-S) score which includes six clinico-pathological variables has been used to predict prostate cancer recurrence [34]. Studies including large, multi-institutional trials have proved that the CAPRA-S score is an effective prognostic tool to predict BCR after radical prostatectomy [35, 36]. To further investigate the role of the eight-lncRNA signature in clinical decision, the signature risk score was evaluated together with the CAPRA-S score. Multivariable Cox regression was used to assess the utility of the eight-lncRNA signature after adjustment for clinical and pathologic variables in GSE21034 cohort. Using the CAPRA-S score as well as its component variables as covariates, we found that the eight-lncRNA signature and the CAPRA-S score were both independent prognostic factors (Table 3). Then, a new score combining CAPRA-S and the eight-lncRNA signature was generated. Addition of the eight-lncRNA signature to CAPRA-S significantly increased its prognostic power: the AUC was 0.77 (95 % CI 0.69–0.86) for CAPRA-S alone compared with 0.82 (95% CI 0.74–0.9) with the addition of our signature (*P* =0.036, Figure 5). Additionally, we also found that the eight-lncRNA signature could stratify men with BCR into those who would or would not develop metastasis (10% of patients with low (only 1) versus 30.7% with high scores developed metastasis).

### Functional characterization of the eight prognostic lncRNAs

**Figure 6:**
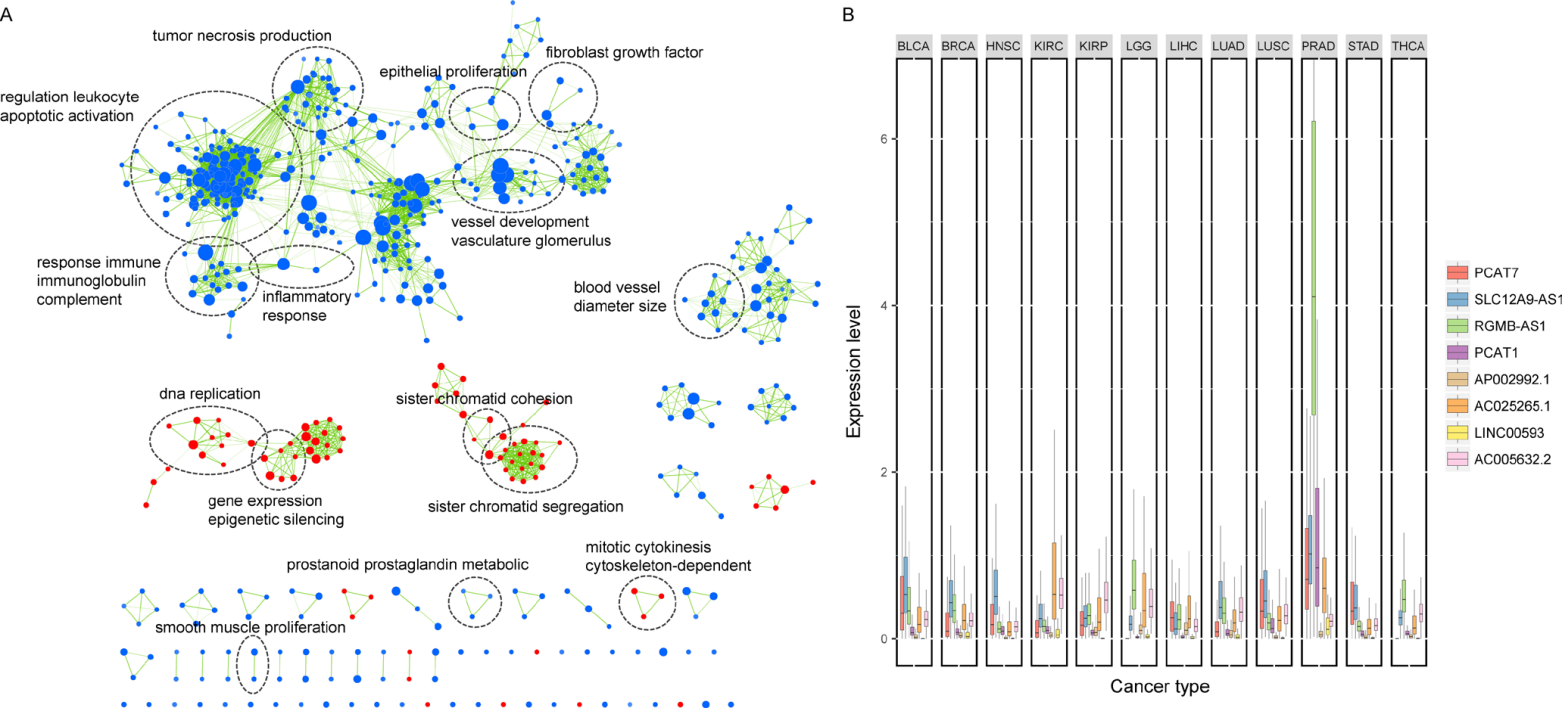
Functional characterization of the lncRNAs in the signature (**A**) Gene set enrichment analysis (GSEA) delineates biological processes correlated with the lncRNA signature. Nodes represent enriched GO terms and an edge represents existing genes shared between connecting GO terms. Nodes are grouped and annotated by their similarity according to related gene sets. (**B**) Box plot comparing the expression of the eight lncRNAs across 12 cancer types. PCAT7, SLC12A9-AS1, RGMB-AS1, PCAT1, AC025265.1 and LINC00593 were highly expressed in prostate cancer across twelve cancers. BLCA, Bladder Urothelial Carcinoma; BRCA, Breast Invasive Carcinoma; HNSC, Head and Neck Squamous Cell Carcinoma; KIRC, Kidney Renal Clear Cell Carcinoma; KIRP, Kidney Renal Papillary Cell Carcinoma; LGG, Brain Lower Grade Glioma; LIHC, Liver Hepatocellular Carcinoma; LUAD, Lung Adenocarcinoma; LUSC, Lung Squamous Cell Carcinoma; PRAD, Prostate Adenocarcinoma; STAD, Stomach Adenocarcinoma; THCA, Thyroid Carcinoma.

Among the eight lncRNAs, two of them (PCAT1 and PCAT7) have been previously revealed to be implicated in PCa [14, 37]. Using the gene set enrichment analysis (GSEA), we identified biological processes associated with the eight-lncRNA signature on the basis of the risk score (FDR < 0.01). As displayed in Figure 6A, our lncRNAs were significantly enriched in cancer-related functions, such as DNA replication, tumor necrosis production and vessel development. We also found that six of the seven risky lncRNAs were specifically highly expressed in prostate cancer across twelve different cancers (Student's *t*-test, FDR < 0.05, Figure 6B), suggesting a cancer-specific pattern of the lncRNA signature. Furthermore, by calculating the Pearson correlations between our prognostic lncRNAs and PCa associated transcriptional factors (TFs), we found that PCAT7, SLC12A9-AS1, RGMB-AS1 and AC005632.2 were significantly correlated with the androgen receptor (AR, *P* value < 0.05), which could regulate the transcription of target genes involved in prostate cell growth, differentiation and apoptosis [38, 39]. Additionally, binding events of AR were found at RGMB-AS1 and the promoter of AC005632.2 by ChIP-sequencing data from the ENCODE project, further suggesting the regulatory relations between these lncRNAs and AR.

## DISCUSSION

During the past years, the discovery of thousands of lncRNAs has provided a new avenue for both diagnosis and prognosis of cancers and other diseases [11, 40]. Evidence from growing publications have demonstrated that the aberrant expressions of lncRNAs can mark the spectrum of disease progression and may serve as independent biomarkers for prognosis in many cancer types [18, 41, 42]. In this study, we comprehensively examined the lncRNA profiles of PCa tissues and paired normal tissues across three independent cohorts from different gene expression measurement platforms and identified an eight-lncRNA signature which was significantly associated with the BCR-free survival. Our use of RNA sequencing and high-density microarrays enabled broad surveillance of lncRNAs. The association between the eight-lncRNA signature and prognosis was robust across all four cohorts in both pooled and individual univariate and multivariate analyses incorporating various clinicopathologic risk factors. More broadly, we found that the eight-lncRNA signature may aid the subclassification of patients based on Gleason score or lymphonodus status and was able to improve on the established clinical algorithm for the risk stratification as well as to reflect the metastatic potential of PCa.

For the eight lncRNAs identified in the signature, PCAT1 has been previously reported to have a potential role in cancer [43, 44]. In agreement with our data, PCAT1 is highly prostate-specific and is upregulated in a subset of high-grade localized and metastatic PCa [45]. PCAT1 induces cell proliferation *in vitro* and functions as a transcriptional repressor by regulating a broad range of genes, including known tumor suppressor genes such as BRCA2 [43]. PCAT1 has been shown to be a target of the Polycomb Repressive Complex 2 (PRC2). The expression of PCAT1 was significantly correlated with that of EZH2 in our PCa patients and knockdown of EZH2 in VCaP caused a dramatic upregulation in PCAT1 expression levels [44]. Another lncRNA, PCAT7, has been shown to be highly expressed in primary and metastatic PCa, and knockdown of it reduces cell growth and soft agar colony formation in LNCaP cells [14]. As for the characteristics of the eight lncRNAs, the functional enrichment analysis showed that they may be involved in the DNA replication, development and proliferation processes. We also pointed that several lncRNAs were significantly correlated with PCa-related TFs. For instance, AC005632.2 was shown to be expression correlated with AR in PCa patients and the binding sites of AR were also localized in the promoter region of AC005632.2. As AR is an important transcription factor in the development and progression of PCa, the association between AR and the lncRNAs may suggest a potential molecular mechanism of these lncRNAs in the development of PCa. Interestingly, we showed that the expression patterns of 87.5% of the lncRNAs in the signature were significantly specific to PCa, with minimal expression in all other tumor types for risky lncRNAs. The tumor specificity of these lncRNAs makes them ideal molecular signatures for clinical utilization and excellent candidates for biomarkers.

By performing multivariable Cox regression analysis, we showed that the prognostic value of the eight-lncRNA signature was independent of traditional clinical and pathological risk factors, including Gleason score and positive LN. Gleason score, or grade of the tumor, has been the single most powerful predictor of PCa prognosis [46]. In the stratified analysis, the eight-lncRNA signature showed prognostic value both in low-grade (Gleason score 2–7) and high-grade (Gleason score≥ 8) patients. Importantly, the eight-lncRNA signature can not only classify patients of the same Gleason grade into high- and low-risk groups but also significantly enhance the prognostic ability of Gleason score, indicating that the signature can improve the accuracy of the prediction of recurrence. As high Gleason score was able to be adopted as one of the criteria for adjuvant radiotherapy or other treatments, our molecular signature should be considered as a factor in selecting patients for adjuvant therapy.

LN-positive prostate cancer indicates an aggressive disease progression. Previous studies reported that the number of positive LNs related to poor BCR-free survival [47, 48]. Identifying prognostic factors for LN-positive PCa is important because patients with a poor prognosis must immediately commence androgen deprivation treatment (ADT) that provides a survival benefit [49]. In our study, we demonstrated that the eight-lncRNA signature was an independent factor to elevate the prognostic power of the number of positive LNs and patients with similar lymphonodus status could be subdivided into high- or low- risk group, indicating that the lncRNA signature may be considered to better stratify patients and facilitate the identification of higher-risk patients who need to be followed up more closely.

We also found that the eight-lncRNA signature was able to improve the accuracy of prediction of BCR on an established clinical algorithm CAPRA-S score [34]. The CAPRA-S score is one of the most accurate clinicopathological models to predict PCa recurrence, and the integration of the lncRNA signature into this model suggesting that assessment of lncRNA signature will be effective in conjunction with the existed prognostic instruments to provide a more accurate prognosis for patients with PCa.

In conclusion, our study presented a powerful lncRNA signature for recurrence of PCa by employing large independent patient cohorts across different gene expression measurement platforms. The eight-lncRNA signature was an independent risk factor of other prognostic factors, supporting the use of it as a potential clinical predictor that could be used alongside standard clinical parameters, such as Gleason score and positive LN. Our results demonstrated that the eight-lncRNA signature may be used to refine the current prognostic models and facilitate further stratification of the PCa patients. Further validation studies in prospective cohorts are required to test the prognostic power and the clinical implications of the signature.

## MATERIALS AND METHODS

### PCa datasets preparation

The transcriptome profiles of 357 samples, including 307 PCa patients and 50 paired adjacent normal tissues were retrieved from the Cancer Genome Atlas (TCGA) prostate adenocarcinoma dataset through the Atlas of Noncoding RNAs in Cancer (TANRIC) [50, 51]. Clinical outcome was evaluated by BCR and patients without recurrence and without the time of recurrence were censored at their respective last day of follow-up. Then, patients without clinical endpoints were removed. Data used for integrative transcriptome analysis were obtained from the ArrayExpress: ERP000550 (RNA-seq) and the Gene Expression Omnibus: GSE29079 (microarray). An external validation data were also included in our study: GSE21034. For microarray data, the Affymetrix Human Exon 1.0 ST Array was used considering its comprehensive coverage of the annotated human lncRNAs. The PCa patients in TCGA were randomly split into a training set (*n* = 184) and an internal validation set (*n* = 123).

### Data processing

The lncRNA annotations were retrieved from GENCODE (v16). We annotated 12,727 and 10,092 lncRNAs in microarray and RNA-seq, respectively. For exon array data, we designed a custom pipeline to re-annotate lncRNAs according to previous studies [14, 52]. The probe sequences were downloaded and uniquely mapped to the human genome (hg19), and probes completely falling into exons of lncRNAs were retained to compute the expression levels of lncRNAs. The raw intensities of the probes were normalized using the RMA normalization method and the expression alterations between tumors and control were calculated by Student's t-test analysis. For sequencing data, sequencing reads were aligned to the human genome (GRCh37/hg19) and read counts for each gene were calculated. Normalized gene expression levels were estimated by FPKM and raw read counts were used to identify differentially expressed lncRNAs by DESeq2 [53]. False discovery rate (FDR) significance level of 5% and fold change of 1.2 were chosen for all differential analyses.

### Identification of lncRNAs associated with PCa recurrence

The association between the lncRNA expression and patient's BCR-free survival was assessed by univariable Cox regression analysis in the training cohort. A risk score formula was then established using the follow formula: Risk score = (0.45666 × expression level of PCAT7) + (0.26201 × expression level of SLC12A9-AS1) + (0.09976 × expression level of RGMB-AS1) + (0.16082 × expression level of PCAT1) + (1.22014 × expression level of AP002992.1) + (0.4591 × expression level of AC025265.1) + (1.6287 × expression level of LINC00593) + (–5.8034 × expression level of AC005632.2), which is a linear combination of the expression levels of the significant lncRNAs weighted by their respective regression coefficients in the univariable Cox regression analysis. With this risk score formula, patients in each cohort were then assigned a risk score and classified into high-risk or low-risk group by the corresponding median risk score.

### Statistical analysis

The survival time of each cohort was estimated by the Kaplan-Meier method, and the survival difference between the high-risk and low-risk groups was compared using the log-rank test. The univariate and multivariate Cox regression models were used to ascertain whether the eight-lncRNA signature was an independent predictor of PCa patient's BCR-free survival. In the models, the normalized expression levels on a log2 scale were used for the four known biomarkers. ROC analysis was performed to compare the sensitivity and specificity of the BCR-free survival prediction based on the eight-lncRNA risk score, Gleason score, positive LN and CAPRA-S score. The combination of two variables was firstly included both as predictors in a logistic regression model and then we used the predictions from the model to plot ROC curves by pROC [54]. Areas under the curve (AUC) were calculated and compared. All statistical analyses were conducted using R program. The significance was defined as *P* values being less than 0.05.

### Functional analyses

We performed functional enrichment analysis for the co-expression relations between protein coding genes and the lncRNA signature on the TCGA cohort using GSEA. FDR *q* value of 1% was used as criteria for significantly enriched gene sets. Cytoscape and the Enrichment Map software were used to visualize the GSEA results [55]. We analyzed the expression profiles of 12 cancers to determine the specificity of the lncRNAs in the signature in prostate cancer. By comparing their expression levels in prostate cancer to those in other cancers, the Student's *t*-test was used to determine statistical significance and the *P*-values were adjusted by FDR. The expression correlations between the prostate cancer-associated transcription factors (TFs) and the eight lncRNAs were computed by Pearson's correlation analysis. TF binding sites were retrieved from ENCODE TFBS ChIP-seq data [56] and the promoter region of lncRNA was defined as 2 kb upstream and 0.5 kb downstream of transcription start site.

## SUPPLEMENTARY MATERIALS FIGURES AND TABLES





## Abbreviations

PCa: prostate cancer
lncRNA: long non-coding RNA
BCR: biochemical recurrence
PSA: prostate-specific antigen
LN: lymph node
HR: hazard ratio
CI: confidence interval
ROC: receiver operating characteristic
AUC: area under the curve
GEO: Gene Expression Omnibus
TCGA: the Cancer Genome Atlas
TANRIC: the Atlas of Noncoding RNAs in Cancer
CAPRA-S: the prostate risk assessment post-surgical
TFs: transcription factors
GSEA: the gene set enrichment analysis
AR: androgen receptor
