# Supplementary material for: Transcriptome analysis reveals a long non-coding RNA signature to improve biochemical recurrence prediction in prostate cancer
Source: Oncotarget. 2018 May 18;9(38):24936–49. doi: 10.18632/oncotarget.25048 (PMC5982764; doi:10.18632/oncotarget.25048)
Supplement: Supplementary file 1 [file oncotarget-09-24936-s001.pdf]

Transcriptome analysis reveals a long non-coding RNA signature to improve biochemical recurrence prediction in prostate cancer

SUPPLEMENTARY MATERIALS

Supplementary Table 1: The 142 consistently altered lncRNAs across the three PCa cohorts . See Supplementary_Table_1

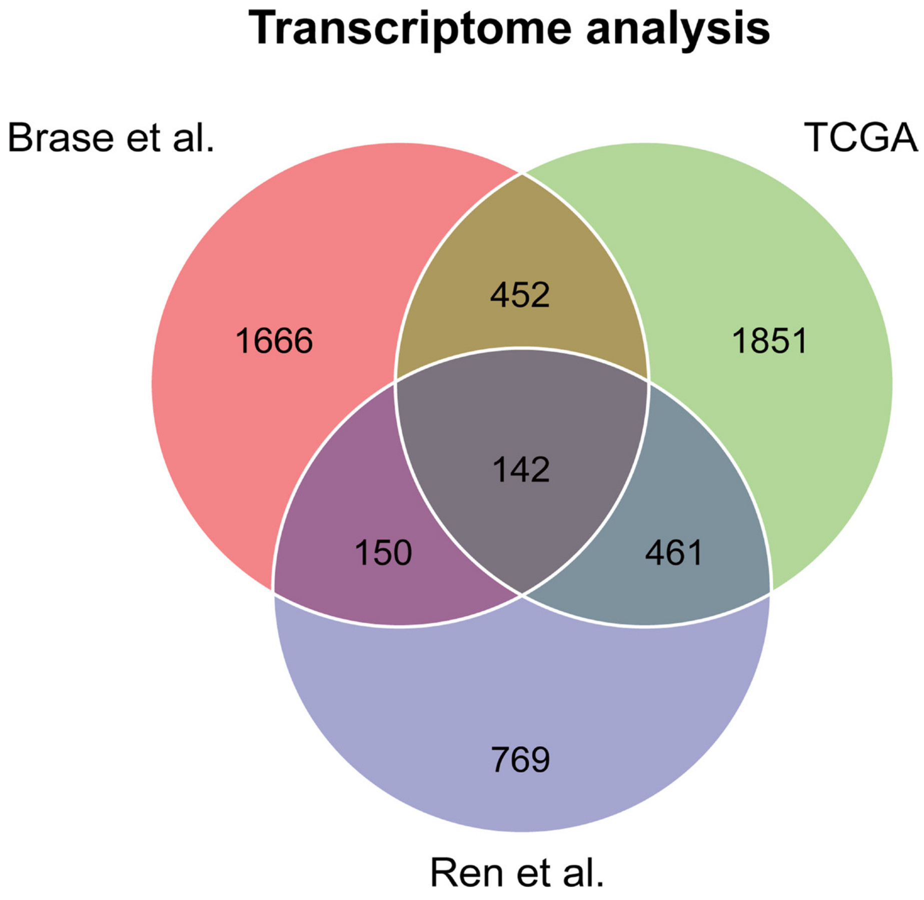

Supplementary Figure 1: Venn diagram showing the intersection of dysregulated lncRNAs across the three PCa cohorts.
